# The influence of competing root symbionts on below‐ground plant resource allocation

**DOI:** 10.1002/ece3.7292

**Published:** 2021-03-02

**Authors:** Christopher A. Bell, Emily Magkourilou, Peter E. Urwin, Katie J. Field

**Affiliations:** ^1^ Faculty of Biological Sciences University of Leeds Leeds UK; ^2^ Department of Animal and Plant Sciences University of Sheffield Sheffield UK

**Keywords:** arbuscular mycorrhiza, competition, mutualism, parasitism, plant‐parasitic nematodes, resource allocation, symbiosis

## Abstract

1. Plants typically interact with multiple above‐ and below‐ground organisms simultaneously, with their symbiotic relationships spanning a continuum ranging from mutualism, such as with arbuscular mycorrhizal fungi (AMF), to parasitism, including symbioses with plant‐parasitic nematodes (PPN).
2. Although research is revealing the patterns of plant resource allocation to mutualistic AMF partners under different host and environmental constraints, the root ecosystem, with multiple competing symbionts, is often ignored. Such competition is likely to heavily influence resource allocation to symbionts.
3. Here, we outline and discuss the competition between AMF and PPN for the finite supply of host plant resources, highlighting the need for a more holistic understanding of the influence of below‐ground interactions on plant resource allocation. Based on recent developments in our understanding of other symbiotic systems such as legume–rhizobia and AMF‐aphid‐plant, we propose hypotheses for the distribution of plant resources between contrasting below‐ground symbionts and how such competition may affect the host.
4. We identify relevant knowledge gaps at the physiological and molecular scales which, if resolved, will improve our understanding of the true ecological significance and potential future exploitation of AMF‐PPN‐plant interactions in order to optimize plant growth. To resolve these outstanding knowledge gaps, we propose the application of well‐established methods in isotope tracing and nutrient budgeting to monitor the movement of nutrients between symbionts. By combining these approaches with novel time of arrival experiments and experimental systems involving multiple plant hosts interlinked by common mycelial networks, it may be possible to reveal the impact of multiple, simultaneous colonizations by competing symbionts on carbon and nutrient flows across ecologically important scales.

## Graphical abstract

Plants resources must be distributed between contrasting, concurrent symbionts. Here, we review and outline major gaps in knowledge surrounding the allocation of plant resources to two contrasting below‐ground root symbionts, the mutualistic arbuscular mycorrhizal fungi, and the parasitic plant parasitic nematodes. Based on recent developments in other symbiotic relationships, we propose hypotheses for the distribution of plant resources between these contrasting below‐ground partners and how this may affect the host.

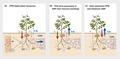

## 1 INTRODUCTION

In nature, plants engage in a variety of complex below‐ground symbioses spanning the mutualistic relationships formed with the near‐ubiquitous arbuscular mycorrhizal fungi (AMF), through to parasitic interactions (Johnson et al., 1997) with pathogenic organisms such as plant‐parasitic nematodes (PPN). These interactions seldom occur in isolation, for instance more than half (51%) of the plant species that play host to PPN are also colonized by AMF, highlighting the prevalence of this tripartite interaction in natural and agro‐ecosystems (FungalRoot [Soudzilovskaia et al., 2020] and Nemabase [Ferris, 2020]). A wealth of other organisms interacts concurrently at the plant:soil interface, such as fungi, oomycetes, bacteria, and nematodes, and exert deleterious or beneficial effects on the plant. These interactions can include beneficial nitrogen fixation (Jacoby et al., 2017) and mineralization of nutrients from the soil for plant uptake (Richardson et al., 2009), whilst others can result in plant disease (Raaijmakers et al., 2009). This greatly affects the structure and function of the soil community, with important, economical implications for crops. In agro‐ecosystems, AMF and PPN both form complex root‐symbiont interfaces that facilitate the transfer of nutrients and will invariably compete for the finite supply of host plant resources in the form of photosynthetically fixed carbon‐based molecules. The same compounds (such as glucose, fructose, and galactose) are often directly acquired by both symbionts (Helber et al., 2011; Rodiuc et al., 2014). However, the mechanisms underpinning resource allocation between competing root symbionts in AMF‐PPN‐plant associations remain unresolved.

Plant parasitism has evolved independently at least four times within the phylum Nematoda (Kikuchi et al., 2017), and there are >4,100 species of PPN from ectoparasitic, semi‐endoparasitic, and endoparasitic groups (Jones et al., 2013). The most damaging are the sedentary endoparasites, which have developed similar, intricate lifestyles to maximize assimilation of plant resources (Bird et al., 2015). The phytophagous ability of PPN has made them one of the four most economically important plant pathogens (Jones et al., 2013), estimated to result in crop yield losses of >US$80 billion per annum (Nicol et al., 2011). Due to their economic importance on key crops worldwide, the majority of research to date has focused on the sedentary root‐knot and cyst nematode species, which we discuss here in relation to their impact on agro‐ecosystems.

AMF, on the other hand, typically confer a variety of potential growth‐enhancing benefits on their plant host (Smith & Smith, 2011). Nutrients that are either beyond the root depletion zone or in an inaccessible form to plant roots may be acquired by AMF and translocated from the soil to host plant roots via an extensive mycelial network, in some cases providing the plant with up to 90% of their phosphorus requirements whilst also contributing toward plant nitrogen and micronutrient needs (Ezawa & Saito, 2018; Field & Pressel, 2018; van der Heijden et al., 2015). The extent of the benefits conferred on plants by AMF is known to vary considerably according to the identities of plant and fungus, as well as various abiotic factors (Field & Pressel, 2018; Johnson et al., 1997). Therefore, it is critical that the impact(s) of environmental factors, including biotic and abiotic, are taken into account when symbiotic function and degree of plant benefits obtained through symbiosis with AMF are considered.

As obligate biotrophs, both PPN and AMF rely on plant‐fixed photoassimilates for nutrition. However, unlike AMF, PPN offer no benefits to their host plant. Instead, PPN typically confer severe impediments on plant growth and productivity (Jones et al., 2013). Despite simultaneous associations with AMF and PPN being common in both agro‐ and natural ecosystems, relatively little is known about how plants cope with these competing root symbionts, especially regarding the allocation of plant resources in crop plants. Here, we do not attempt to review AMF‐PPN‐host interactions in their entirety, rather we highlight the currently ill‐defined key factors that may regulate the distribution of host plant resources to the competing symbionts. To instigate new ideas and research into this area, we hypothesize alternate scenarios for nutrient allocation within tripartite interactions between plants, AMF, and PPN and the potential outcomes for each. By defining this area, a more holistic understanding of complex nutrient allocation at the plant–soil interface may be reached, with considerations of impacts on plant defenses and the potential exploitation of AMF as biocontrol agents to enhance sustainable agro‐ecosystems.

## 2 EFFECTOR MOLECULES: THE COMMON DENOMINATOR IN THE ESTABLISHMENT OF SYMBIOSIS

In order to derive their maximum benefit, plants must discriminate between symbionts that are mutualistic and those that are parasitic. The recognition of pathogen‐associated molecular patterns (PAMPs) by plant pattern recognition receptors (PRR) may allow plant hosts to differentiate potentially mutualistic symbionts from pathogenic organisms that invariably induce a stronger plant defense response (Teixeira et al., 2019). Plants initiate similar defense chemistry cascades in response to both mutualistic and pathogenic interactions (Cameron et al., 2013). However, there are key differences between plant responses initiated in response to mutualists compared to those triggered by pathogens. Mutualist‐triggered responses permit entry to plant tissues but restrict the organism from overwhelming the host plant, whereas the pathogen‐triggered response restricts any such entry (Plett & Martin, 2018).

Before the establishment of symbiosis, sedentary PPN secrete a wide array of effector molecules that may associate with host proteins to promote colonization of host roots (Rai et al., 2015), suppress plant defense responses (Chen et al., 2018) and reprogram host cells to form specialized feeding sites, which increase metabolic activity and accumulate plant resources (Rodiuc et al., 2014). Synthesis of these molecules represents a large energy investment by PPN to promote successful hijacking and redirection of plant photoassimilates. Similarly, AMF secrete a range of effectors that facilitate the establishment of a mutualistic relationship with the plant, many of which are conserved between AMF species, suggesting their involvement in core symbiotic processes (Lanfranco et al., 2018). However, in contrast to PPN systems, relatively little is known about AMF effectors. This represents an area of intense research and rapid development, with recent findings suggesting some similarity between the two systems. For example, similar to PPN, AMF effectors appear to be expressed on a host‐specific basis (Bell et al., 2019; Lanfranco et al., 2018) and can thus interfere with plant defense responses (Chen et al., 2018; Kloppholz et al., 2011). This may provide a mechanistic basis for the observed differences in nutritional exchanges between AMF and host plants (e.g., Field et al., 2017) as a result of species‐specific variation in resource allocation into defense chemistry (Cameron et al., 2013).

As both AMF and PPN express effector molecules that suppress plant defense responses and initiate the formation of symbiotic structures within root tissues, it is possible that these molecules could also mediate crosstalk between symbionts. Such crosstalk would be likely to affect the simultaneous formation of both symbioses, either facilitating their establishment within the root or initiating a localized “arms race” to determine the order of symbiont establishment. The success or speed of establishment within the root is likely to be critical, being key to determining the capability of the symbiont to acquire resources from the host and subsequently develop. It is possible that the arrival of one symbiont may impede the second and thereby restrict their resource flow. Alternatively, they may co‐exist together in the same region of root, with the first to arrive even facilitating establishment of the second. A repertoire of unknown effector molecules may exist that interact and/or interfere with establishment of other root symbionts, such as nitrogen‐fixing bacteria or other root pests and pathogens. For the nematode, establishment of the feeding site via effectors is well‐documented (Mejias et al., 2019). In resistant hosts, the recognition of such effectors is suggested to trigger cell death, shutdown of the feeding site and subsequent induction of nematode mortality (Postma et al., 2012). There is clear potential for AMF‐PPN interactions to affect the formation of the PPN feeding site, either through competition for space within the root cells or through changes in plant defense chemistry, including perception and actions of PPN effectors. As AMF colonization of root cells does not induce programmed cell death in the way PPN colonization does, the signaling and recognition mechanisms used in plant–AMF interactions may either inhibit or promote plant defense responses against concurrent nematode invaders. More research is needed into effectors, both specific and general, in both AMF and PPN to promote our knowledge of the structure, function, and interactions between these important symbiotic signaling molecules and their influence on wider plant–rhizosphere processes.

## 3 COMPETITION FOR SPACE AND RESOURCES WITHIN THE ROOT

As well as producing effector molecules that influence the establishment of symbioses and acquisition of resources, AMF and PPN frequently occupy the same space within host plant roots, therefore competition for plant resources within the root is likely to be acute (Jung et al., 2012). The intensity of competition between symbionts will vary depending on the precise location of PPN feeding sites and intracellular AMF structures, and the relative resource sink strengths of the PPN (Carneiro et al., 1999) and AMF species (Thirkell et al., 2020) present. The time of arrival of each symbiont is also likely to have a profound effect (Werner & Kiers, 2015) on the allocation of plant resources, which is particularly pertinent in the case of colonization by PPN and AMF, as both respond to chemical cues secreted from plant roots (Lanfranco et al., 2018; Sikder & Vestergård, 2020). Plant hormones, such as strigolactones, have been shown to promote symbiosis with AMF by initiating fungal spore germination and stimulating hyphal branching (Akiyama et al., 2005). Strigolactones have also been shown to both enhance (Lahari et al. 2019) and reduce (Xu et al., 2019) root‐knot nematode infection in rice and tomato plants, respectively. The role of strigolactone signals in dual AMF and PPN symbioses and how these cues may differ chemically and/or functionally according to symbiont perception remains unexplored.

Despite differences in signal perception and plant defense responses, AMF and PPN frequently colonize the same plant root systems (Ferris, 2020; Soudzilovskaia et al., 2020). It is therefore critical to plants hosting simultaneous AMF and PPN symbionts that they modulate resource allocation in order to promote the mutualistic interactions with AMF whilst minimizing resource losses to PPN. Discrimination between “generous” and “uncooperative” AMF mutualists is reported in plants, whereby AMF that provide ample mineral nutrients to the host plant are rewarded with photosynthates whilst those that withhold nutrients are not (Kiers et al., 2011). Although the precise mechanisms remain under debate (Kiers et al., 2016; Walder & van der Heijden, 2015), similar mechanisms of discrimination could be at play in multi‐symbiont associations, with the host plant being able to detect and effectively shut down resource allocation to symbionts perceived to impart little or no benefit whilst promoting allocation to mutualists. The benefits of AMF–plant symbioses have been reported to outweigh the detrimental impacts of colonization by PPN and the presence of AMF can lead to reduced nematode infection, although evidence of this effect is often equivocal (e.g., Garita et al., 2019; De La Peña et al., 2006; Vos et al., 2012). Increased resistance or tolerance to the pathogen conferred to the plant by AMF may be a result of heightened nutrient status of the host (Schouteden et al., 2015), although this could conversely simultaneously increase plant attractiveness to pests such as herbivores (Charters et al., 2020).

**FIGURE 1:**
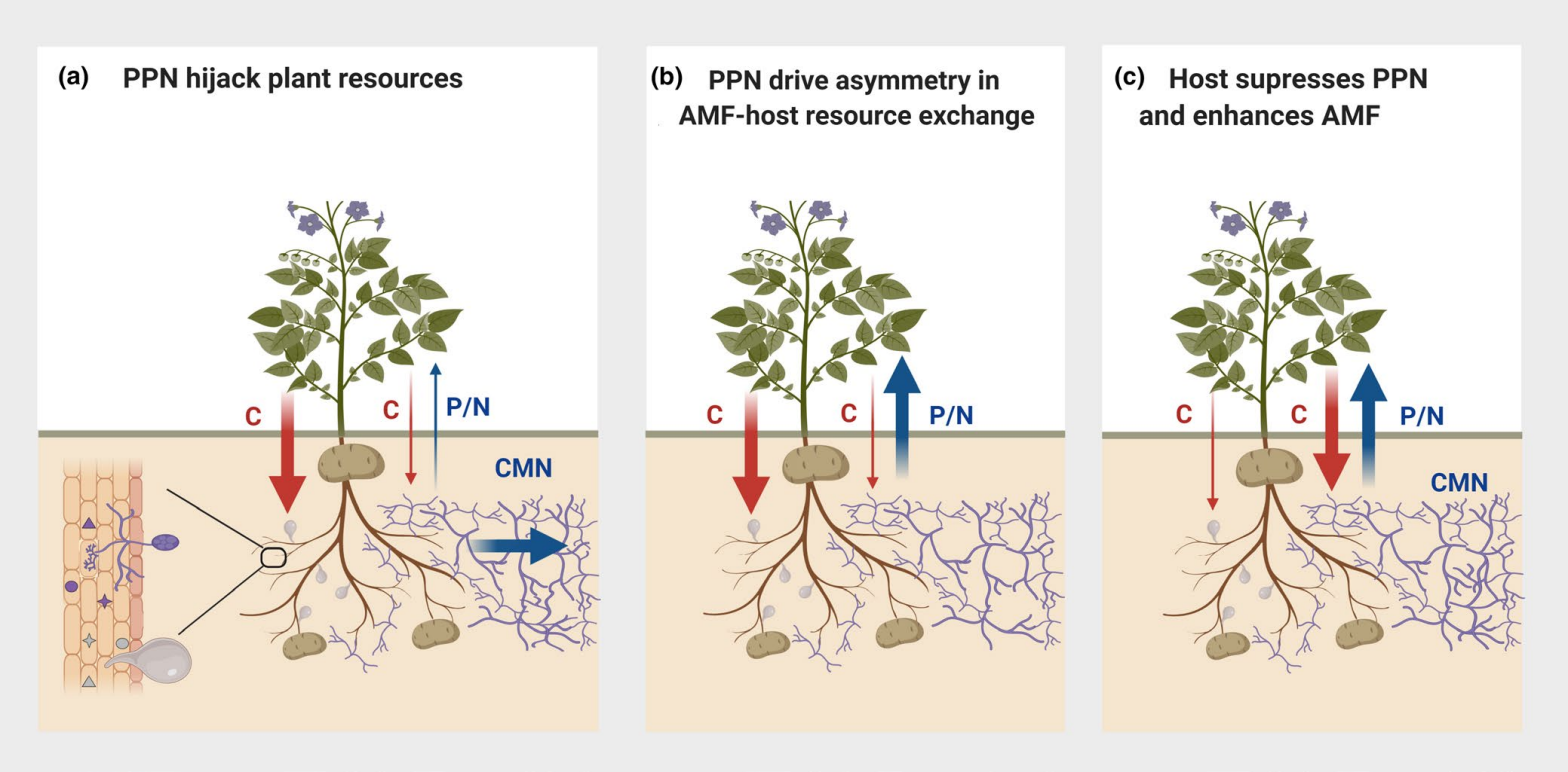
Alternative scenarios for the allocation of resources in the PPN‐AMF‐plant tripartite symbiosis. (a) Plant‐parasitic nematodes (PPN) acquire the majority of the carbon‐based plant resources (C), and the arbuscular mycorrhizal fungi (AMF) receive a reduced allocation. In turn, this leads to diminished transfer of nutrients from AMF to the host (P/N), whilst the AMF utilize the common mycelial network to translocate nutrients toward other hosts in the system. (b) Identical carbon allocation to symbionts as described in (a), with the majority acquired by the PPN. AMF‐derived nutrients continue to be transferred to the host even though there are reduced resources exchanged. (c) The host plant may distinguish PPN offer no reciprocal benefit and reduce delivery of C. In turn, resources are directed toward more beneficial AMF. In each scenario, the resource allocation to each symbiont may be regulated by the host or through local effector crosstalk within feeding structures (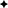
●▲, a). Red arrows—host to symbionts carbon flow; Blue arrows—AMF to host phosphorus/nitrogen flow; width of arrows—the strength of the flow; CMN—common mycelial network. Created with BioRender.com

Upon colonization, PPN modify host roots to enable feeding, resulting in the redirection of substantial plant resources toward the parasite for ingestion (Hofmann & Grundler, 2007), alongside suppression of host plant photosynthesis (Hofmann & Grundler, 2007; Wang et al., 2019). As a result of these changing strengths of plant carbon sinks and sources, it is likely that colonization by PPN influences the flux of resources toward the mutualist, resulting in impacts on the myriad of host plant benefits offered by AMF. Here, we propose three potential outcomes on resource allocation as a result of the AMF‐PPN symbioses with the host (Figure 1):

1. Direct acquisition of plant carbon resources by PPN may reduce the allocation of such resources toward AMF partners. In turn, this reduces transfer of nutrients from AMF to the host plant causing the host to lose resources both directly via parasitism, and indirectly via loss of nutrient income through reduced mutualistic interactions with AMF (Figure 1a).
2. The majority of plant‐carbon is allocated to the parasite whilst AMF‐acquired nutrients continue to flow into the roots, possibly being ultimately redirected toward the parasite (Figure 1b). In a similar fashion to recently described AMF‐aphid‐host systems (Charters et al., 2020), feeding by PPN may induce asymmetrical carbon‐for‐nutrient exchange between mycorrhizal symbionts, with PPN‐infested plants transferring dramatically less carbon to AMF partners but nutrient transfer from AMF to plant is maintained. In this scenario, it is likely that distribution of carbon across a common mycelial network between neighboring plants (Whiteside et al., 2019) provides sufficient carbon resources for AMF to maintain nutrient transfers to plants despite reduced host contribution. This may underpin the greater success some nematode species appear to have on plants that are co‐colonized by AMF (Frew et al., 2018).
3. The host distinguishes symbionts that offer no return benefit and effectively shut down delivery of photosynthates to these regions (Figure 1c), thereby limiting carbon wastage, as previously shown in unproductive legume root nodules (Kiers et al., 2003). In this scenario, the PPN would acquire fewer resources, compared to when infecting roots not colonized by AMF. The nematode may then develop slower or produce fewer eggs due to the resources partitioned to the mutualistic fungus, which can then develop.

In the field, common mycelial networks (CMN) allow for communication and distribution of resources between multiple plants and mycorrhizal fungi within communities. CMNs may move nutrients from richer regions in the soil where resources are abundant, to regions of poorer nutrient status, where associations with AMF are promoted (Whiteside et al., 2019). This is likely to also be the case in agro‐ecosystems, although it has been shown that the diversity of AMF that could form such networks is diminished as a result of conventional agricultural soil management practices (Daniell et al., 2001; Helgason et al., 1998). If a plant, or group of plants, within a CMN is infected with PPN, AMF may shut down nutrient allocation to those hosts, and instead translocate nutrients toward other hosts, thereby ensuring resource supply to maintain the integrity and function of the CMN.

Alternatively, the competition for plant resources may be driven by the nutritional requirements of each symbiont rather than their sink strength or molecular arsenals. This may vary greatly between different mutualistic and parasitic symbionts. Each of the hypotheses described above assumes concurrent colonization by AMF and PPN at a given time point, however the dynamics of the system are likely to be greatly influenced by the timing of arrival of each symbiont, as well as the phenology of the host plant. For example, mycorrhizal colonization typically results in primed plant defenses, which can reduce impacts of parasites (Jung et al., 2012), meaning that nematodes arriving to the plant after mycorrhizal colonization must face and overcome pre‐acquired mycorrhiza‐induced resistance (Cameron et al., 2013) before establishing a symbiosis and accessing plant resources. Furthermore, multiple species and individuals of both AMF and PPN may colonize the same host, with the arrival time of the different species/individuals likely to have a large impact on the host and its resources. For example, it is unknown whether or not the first nematode to invade a host root will have greater access to resources than subsequent individuals of the same or different species. Availability of plant resources for symbionts will vary according to seasonal changes in the environment and host plant growth stage as a result of changes in plant physiology (Idoia et al., 2004). It is possible that biochemical crosstalk via effectors or other symbiont‐derived molecules influences the allocation of available plant resources to co‐colonizing symbionts. If AMF are able to maintain a supply of nutrients to the host without those nutrients being redirected and ingested by the parasite, then the enhanced nutrient status of host plants may drive the positive impact of AMF on the growth of nematode‐infected plants (Garita et al., 2019).

In studying plant symbioses, it is crucial that we consider the holistic impact of the myriad of symbionts that may co‐occur and interact at any time, rather than studying a single chosen symbiotic interaction in isolation. Here, we have described the impact of an important plant parasite on the mutualistic AMF–host relationship and hypothesized several outcomes on host resource allocation, upon which to experiment. Other investigations into tripartite systems have made use of well‐established methods in isotope tracing and nutrient budgeting to address‐related questions regarding plant resource allocation (Charters et al., 2020). Coupled to other experimental approaches, including time of arrival and multiplant systems, similar approaches may be applicable here to dissect the physiological mechanisms and responses integral to AMF‐PPN‐host symbioses. This is particularly pertinent within agro‐ecosystems where the plant defense‐promoting properties of plant colonization by AMF are often touted (e.g., Cameron et al., 2013) but seldom tested in complex, multisymbiotic scenarios. A better understanding of these mechanisms in terms of nutrient flows and regulation of colonization may lead to exploitation of AMF via wider (and better informed) usage of commercial inocula, or indeed their avoidance due to undesirable effects on other organisms, such as the potential for AMF to elevate the nutrient status of the host thereby offering more to the feeding nematode population (Frew et al., 2018).

## 4 CONCLUSION

Plant roots are integral to the concept of an ecosystem, existing as critical components of below‐ground systems that underpin those above‐ground, rather than as single entities interacting with individual symbionts. Within this system, the complex interactions between multiple below‐ground symbionts in competition for plant resources remain critically under‐explored, resulting in a number of important, outstanding research questions such as: how is allocation of plant resources regulated in the AMF‐PPN‐host system? Does regulation occur at the site of competition, systemically within the single host, or is it modulated by the common mycelial network across multiple plants? By addressing these, a more holistic understanding of contrasting tripartite below‐ground interactions may be acquired that may allow us to evaluate the roles of AMF in the field and the influence on plant community, and subsequently ecosystem, structure, and function. Tracking the movement of resources in plants with different combinations of root symbionts will help to determine the consequences of PPN infection for plant–AMF associations, potentially also helping to explain the disparity between the species‐specific effects of different AMF and nematode species on AMF biocontrol efficacy and the role(s) of common mycelial networks in agro‐ecosystems. The mutualistic AMF and parasitic PPN symbioses discussed here represent the granularity of the below‐ground ecosystem. There are a huge number of other organisms that interact at the root–soil interface, with each component of the soil community impacting each other either directly or indirectly. As there are finite plant resources available that the below‐ground community is in competition for, particularly in agri‐ecosystems, the influence of such interactions on plant resource allocation must be understood in order that it may be managed to promote beneficial interactions and optimum plant growth conditions. By investigating such granularities of the below‐ground ecosystem, future research will provide a platform from which to begin to unpick and understand other complex, multitrophic interactions that exist in nature and thereby promote a fuller, more holistic understanding of the rhizosphere.

## CONFLICT OF INTEREST

None declared.

## AUTHOR CONTRIBUTION

**Christopher A. Bell:** Conceptualization (equal); Formal analysis (equal); Investigation (lead); Methodology (equal); Writing‐original draft (equal); Writing‐review & editing (equal). **Emily Magkourilou:** Conceptualization (equal); Investigation (equal); Methodology (equal); Writing‐original draft (equal); Writing‐review & editing (equal). **Peter E. Urwin:** Conceptualization (equal); Funding acquisition (equal); Project administration (equal); Supervision (equal); Writing‐review & editing (equal). **Katie J. Field:** Conceptualization (equal); Funding acquisition (equal); Project administration (equal); Supervision (equal); Writing‐review & editing (equal).

## Data Availability

No data.
